# The effect of seminal plasma β-NGF on follicular fluid hormone concentration and gene expression of steroidogenic enzymes in llama granulosa cells

**DOI:** 10.1186/s12958-019-0504-9

**Published:** 2019-07-22

**Authors:** Ximena P. Valderrama, Jose F. Goicochea, Mauricio E. Silva, Marcelo H. Ratto

**Affiliations:** 1 Department of Animal Science, Faculty of Veterinary Sciences, Universidad Austral de Chile, Valdivia, Chile; 2 Department of Animal Reproduction and Surgery, Faculty of Veterinary Medicine and Zootechnics, Universidad Nacional Hermilio Valdizán, Huánuco, Peru; 3 College of Veterinary Medicine, Faculty of Natural Resources, Universidad Catolica de Temuco, Temuco, Chile

**Keywords:** Llama, OIF, β-NGF, Preovulatory follicle, Corpus luteum, Steroidogenic enzymes and VEGF gene expression

## Abstract

**Background:**

Nerve growth factor (β-NGF) from llama seminal plasma has been described as a potent ovulatory and luteotrophic molecule after intramuscular or intrauterine infusion in llamas and alpacas. We tested the hypothesis that systemic administration of purified β-Nerve Growth Factor (β-NGF) during the preovulatory stage will up-regulate steroidogenic enzymes and Vascular Endothelial Growth Factor (VEGF) gene expression in granulosa cells inducing a change in the progesterone/estradiol ratio in the follicular fluid in llamas.

**Methods:**

*Experiment I:* Female llamas (*n* = 64) were randomly assigned to receive an intramuscular administration of: a) 50 μg gonadorelin acetate (GnRH, Ovalyse, Pfizer Chile SA, Santiago, Chile, *n* = 16), b) 1.0 mg of purified llama β-NGF (*n* = 16), or c) 1 ml phosphate buffered saline (PBS, negative control group, *n* = 16). An additional group of llamas (*n* = 16) were mated with a fertile male. Follicular fluid and granulosa cells were collected from the preovulatory follicle at 10 or 20 h after treatment (Time 0 = administration of treatment, *n* = 8/treatment/time point) to determine progesterone/estradiol concentration and steroidogenic enzymes and VEGF gene expression at both time points. *Experiment II:* Granulosa cells were collected from preovulatory follicles from llamas (*n* = 24) using ultrasound-guided transvaginal follicle aspiration for in vitro *culture* to determine mRNA relative expression of Steroidogenic Acute Regulatory Protein (StAR) and VEGF at 10 or 20 h (*n* = 4 replicates) and progesterone secretion at 48 h (*n* = 4 replicates) after LH or β-NGF treatment.

**Results:**

*Experiment I:* There was a significant increase in the progesterone/estradiol ratio in mated llamas or treated with GnRH or purified β-NGF. There was a significant downregulation in the mRNA expression of Aromatase (CYP19A1/P450 Arom) for both time points in llamas mated or treated with GnRH or llama purified β-NGF with respect to the control group. All treatments except β-NGF (20 h) significantly up regulated the mRNA expression of 3-beta-hydroxysteroid dehydrogenase (HSD3B) whereas the expression of StAR and Side-Chain cleavage enzyme (CYP11A1/P450scc) where significantly up regulated only by mating (20 h), or β-NGF at 10 or 20 h after treatment. VEGF was up regulated only in those llamas submitted to mating (10 h) or treated with purified β-NGF (10 and 20 h). *Experiment II:* Only β-NGF treatment induced an increase of mRNA abundance of StAR from llama granulosa cells at 20 h of in vitro culture. There was a significant increase on mRNA abundance of VEGF at 10 and 20 h of in vitro *culture* from granulosa cells treated with β-NGF whereas LH treatment increases VEGF mRNA abundance only at 20 h of in vitro culture. In addition, there was a significant increase on progesterone secretion from llama granulosa cells 48 h after LH or β-NGF treatment.

**Conclusions:**

Systemic administration of purified β-NGF from llama seminal fluid induced a rapid shift from estradiol to progesterone production in the preovulatory follicle. Differences in gene expression patterns of steroidogenic enzymes between GnRH and mated or β-NGF-treated llamas suggest local effects of seminal components on the preovulatory follicle.

## Background

Mating-induced ovulation has been documented in several species of Camelidae family, such as, llamas [1, 2], alpacas [1, 3–5] and Bactrian camels [6]. However, studies conducted in llamas and alpacas [7–10] as well as in Bactrian camels [11, 12] have documented the presence of a potent ovulation-inducing factor (OIF) in the seminal plasma of these species, challenging the concept of physical induced ovulation. We have developed an alpaca and llama model to determine the presence of this factor in the seminal plasma from species with both induced and spontaneous ovulation [7–9, 13, 14]. The factor has been biochemically and functionally characterized during the last 18 years [7, 10, 15–20]. In a study involving peptide sequencing and X-ray diffraction, OIF purified from llama seminal plasma was found to have the identical sequence and protein structure as nerve growth factor [21].

Based on previous studies, β-NGF from llama seminal plasma has been described as a potent ovulatory molecule (i.e. induces the secretion of pituitary LH and ovulation) which also has a luteotrophic effect after intramuscular or intrauterine infusion in llamas and alpacas [7, 9]. The observed effect of seminal plasma on the function of the ensuing corpus luteum (CL) has been surprising and no less significant than its effects on ovulation. The CL developed after ovulations induced by seminal plasma treatment tended to be larger and regressed later than the CL resulting from GnRH treatment, producing twice as much progesterone [7]. The positive relationship between the magnitude of LH release during the preovulatory surge and subsequent luteal form and function in females llamas treated with homologous seminal plasma vs GnRH provides rationale for the hypothesis that the luteotrophic effect of β-NGF is mediated by LH. In this regard, the LH-releasing and luteotrophic effects of seminal plasma have been confirmed in three subsequent studies using β-NGF isolated and purified from the seminal plasma of llamas [10, 16, 22].

Moreover, in a recent llama study [23] the increase of the vascular area of the CL was highly correlated to progesterone production in females treated with β-NGF. Similarly, Fernandez et al. [24] have documented that the highest luteotrophic effect was observed when llamas were given two doses of 1 mg of purified β-NGF before and during the time of ovulation, as opposed to a single dose before ovulation. However, along with the LH effect we cannot rule out a local effect of llama seminal plasma β-NGF at the ovarian level that could potentiate progesterone production. Indeed, the luteotrophic effect of β-NGF was associated with enhanced tissue vascularization during the preovulatory period and early stages of CL development enhancing steroidogenesis [23, 24].

The preovulatory LH surge is the key signal for inducing not only ovulation but also is determinant for the initial stage of luteinization and CL formation and functioning. In spontaneous ovulators such as bovine, the main events associated with final follicle differentiation are acquisition of LH receptors in granulosa cells [25] and an acute increase in mRNA expression of steroidogenic enzymes: steroidogenic acute regulatory protein (StAR), cytochrome P450 side-chain cleavage (P450scc or CYP11A1), cytochrome P450 aromatase (P450 aromatase or CYP19A1) and 3β -hydroxysteroid dehydrogenase (HSD3B). Of those, StAR, HSD3B1 and P450scc are present in both theca and granulosa cells [26]. In this sense, the preovulatory LH peak is responsible for follicular steroidogenesis shift from predominantly estrogen/androgen to progesterone production during the periovulatory period. Interestingly, in a previous llama study [27], the systemic administration of β-NGF did increase Corpus Luteum vascularization and up regulated the expression of CYP11A1/P450scc and StAR transcripts enhancing progesterone secretion during the early luteal phase. However, no information is available regarding the effect of LH peak and/or seminal components as β-NGF on follicular fluid hormone concentration and steroidogenic enzyme and angiogenic factors, such as VEGF gene expression from preovulatory follicles in llamas.

We believe that systemic administration of purified β-NGF will induced an up regulation of steroidogenic enzymes and angiogenic growth factor gene expression resulting in a change in the ratio of progesterone/estradiol (P4/E2) in the follicular fluid during the preovulatory stage in llamas.

## Methods

Experimental procedures were reviewed and approved by the Universidad Austral de Chile Bioethics Committee and were performed in accordance with the animal care protocols established by the same institution.

### Semen collection and protein purification

Semen was collected at the Animal Reproduction Laboratory, Universidad Austral de Chile, Valdivia, Chile (39° 38′S - 73° 5′W and 19 m above sea level) from five mature male llamas, twice per week for 5 months before the start of the experiment. Semen was collected using a sheep artificial vagina as previously described [7]. Each ejaculate was diluted 1:1 (vol/vol) with phosphate buffered saline (PBS, GIBCO, Grand Island, NY, USA) and centrifuged for 30 min at 1500 g at room temperature. A pool of sperm-free seminal plasma was stored at − 20 °C. Purification of β-NGF was performed in a two-step procedure, as previously described [10, 12]. In brief, seminal plasma was loaded into a type 1 macro-prep ceramic hydroxylapatite column (1 cm × 10 cm, 40 μm, Bio-Rad Laboratories, Hercules, CA, USA) previously equilibrated with 10 mM sodium phosphate, pH 6.8, and flow rate of 0.5 mL/min. An eluted fraction showing a major protein on SDS-PAGE was concentrated in PBS (pH 7.4) using a 5 kDa cut-off membrane filter device (Vivaspin, Sartorius, Göttingen, Germany) and subsequently loaded onto a gel filtration column (SEC, hi Prep 26/60 Sephacryl S-100, Amersham Laboratories, Piscataway, NJ, USA). The purification procedure was carried out at room temperature at a flow rate of 0.5 ml/min using fast protein liquid chromatography (FPLC, Amersham Laboratories, Piscataway, NJ, USA). Elution was performed isocratically using PBS at pH 7.4. The bioactive fraction after gel filtration was identified using an in vivo llama ovulation bioassay [15].

### Experiment 1: in vivo *study*

#### Animals and treatment groups

Non-pregnant, non-lactating female llamas (*n* = 64) ≥4 years of age and weighing 100 to 120 kg, were used during May to June at the Kotosh Research Station, Universidad Hermilio Valdizan in the Department of Huánuco, Peru (8° 21` 47S - 76° 18` 56 W and 1,800 m above sea level). Llamas were maintained in separate pens, and had access to natural pasture supplemented with hay and water ad libitum*.* The ovaries of the llamas were examined daily by transrectal ultrasonography using a 7.5 MHz linear-array transducer (Aloka, SSD-500, International Clinics, Santiago, Chile). When a growing follicle ≥8 mm in diameter was detected [28], llamas were randomly assigned to receive an intramuscular administration of: a) 50 μg gonadorelin acetate (GnRH, Ovalyse, Pfizer Chile SA, Santiago, Chile, *n* = 16), b) 1.0 mg of purified llama β-NGF (*n* = 16), or c) 1 ml phosphate buffered saline (PBS, negative control group, *n* = 16). An additional group of llamas (*n* = 16) were mated once with a fertile male.

#### Follicular fluid and granulosa cell collection

Ovaries of llamas were collected after slaughter in the local abattoir and the preovulatory follicle was identified and isolated. Follicular fluid and granulosa cells were collected at 10 and 20 h after treatment (Time 0 = administration of treatment, *n* = 8/treatment/time point). Follicular fluid was aspirated from the preovulatory follicles and stored at − 20 °C until assayed for progesterone and estradiol concentration. After aspiration, follicles were bisected and granulosa cells were removed by scrapping the internal surface of the follicular wall with a sterile Pasteur pipette into a petri dish with PBS medium. Cells were collected by centrifugation at 400 x g for 10 min, frozen in liquid nitrogen and stored at -80 °C until mRNA was isolated.

The 10 and 20 h sampling schedule established in the present study was based on the preovulatory LH profile previously described in llamas [10, 16, 17, 22], in which an LH surge peaked consistently after 2 h of mating or GnRH or β-NGF administration (Time 0 = treatment). Additionally, ovulation was observed to occur 26–28 h after treatment administration. Therefore, it was expected that changes in gene expression would be detectable between 8 and 18 h after LH peak maximum concentration, corresponding to 10 and 20 h of granulosa cell collection from initiation of treatment.

#### Follicular fluid hormone concentration

Follicular fluid progesterone concentration was determined by solid-phase radioimmunoassay kit (Progesterona RIA-CT (KIP1458) DIASource ImmunoAssays S.A. Louvain-la-Neuve, Belgium), as previously reported [16–18]. The intra-assay coefficient of variation was 0.37–3.97%, the minimum detectable limit 0.05 ng/ml, coefficient of variation of internal standard < 3.99%, the limit of quantification observed for the assay was 0.47 ng/ml. Follicular fluid estradiol concentration was determined by solid-phase radioimmunoassay kit (Pantex Estradiol 100 μl direct 125I (catalog n. 174.1) Santa Monica, California, USA). The intra-assay coefficient of variation was 1.24–4.56%, the minimum detectable limit 10 pg/ml, coefficient of variation of internal standard < 2.99%, the limit of quantification observed for the assay was 7.92 pg/ml.

### Experiment II: in vitro *study*

#### Primary culture of llama granulosa cells

Non-pregnant, non-lactating female llamas (*n* = 12) ≥4 years of age and weighing 90 to 136 kg, were used during April to May at the Animal Reproduction Laboratory, Universidad Austral de Chile, Valdivia, Chile (39° 38′S - 73° 5′W and 19 m above sea level). Llamas were submitted to transvaginal ultrasound-guided follicle ablation of all ovarian follicles ≥5 mm using a 19-guage needle attached to a 5 MHz convex-array transducer to synchronize follicular wave emergence, as described previously [29]. Granulosa cells (GC) were collected by flushing the preovulatory follicle by transvaginal ultrasound-guided follicle aspiration using a 5.0 MHz convex-array ultrasound transducer coupled to a 19-gauge needle as described previously [29–31]. The follicular fluid collected from an individual female was centrifuged at 400 x g for 10 min. The cell pellet was then re-suspended in 2 ml of Ham’s F-12/DMEM (Life Technology, USA) and centrifuged again at 1200×g for 45 min in a 3 ml 50% Percoll (Sigma, USA) column to separate GC from erythrocytes and interstitial cells [32]. Purified GC were collected from the top of the Percoll column and washed twice by centrifugation at 400 x g for 6 min. Because a low number of granulosa cells was collected from individual animals using transvaginal follicle aspiration, it was necessary to pool three animals after Percoll purification to get one biological replicate (*n* = 4 independent replicate culture). Granulosa cells were plated into 24-well culture plates (at 1 × 10^5^ cells/well) in Ham’s F-12/DMEM (Sigma, USA) supplemented with 10% fetal bovine serum (Sigma, USA), and antibiotics (penicillin/streptomycin and gentamicin, Sigma, USA) and incubated in an atmosphere of 95% air, 5% of CO_2_ at 38 °C and high humidity for 48 h. After 80% of confluence, medium was replaced with serum-free Ham’s F-12/DMEM and cells were treated with 30 ng/ml of LH or 50 ng/ml of llama-purified β-NGF for 10 or 20 h. For RNA-isolation, Trizol reagent (Invitrogen, life Technology, USA) was added to the cells at the end of the in vitro culture and extraction followed the manufacturer’s protocol.

#### Progesterone secretion from primary culture of llama granulosa cells

Non-pregnant, non-lactating female llamas (*n* = 12) ≥4 years of age and weighing 90 to 136 kg, were used during April to May at the Animal Reproduction Laboratory, Universidad Austral de Chile, Valdivia, Chile (39° 38′S - 73° 5′W and 19 m above sea level). Llamas were submitted to the similar follicular synchronization and aspiration procedure as previously described (above). In addition, granulosa cells were processed similar to previously described (above). After Percoll purification, a pool of three animals was necessary to achieve one biological replicate (*n* = 4 independent replicates culture). Granulosa cells were plated into 24-well culture plates (at 1 × 10^5^ cells/well) in Ham’s F-12/DMEM (Sigma, USA) supplemented with 10% fetal bovine serum (Sigma, USA), and antibiotics (penicillin/streptomycin and gentamicin, Sigma, USA) and incubated in an atmosphere of 95% air, 5% of CO_2_ at 38 °C and high humidity for 48 h. After more than 80% of confluence, the medium was replaced with serum-free Ham’s F-12/DMEM and cells were treated with 30 ng/ml of LH or 50 ng/ml of llama-purified β-NGF for an additional 48 h of in vitro culture. After that incubation period, the media was collected and stored at -20 °C until assayed for progesterone. Progesterone concentration was measured as previously described above for follicular fluid concentration.

### RNA isolation and real time PCR (Q-PCR) analysis

Total RNA was extracted from granulosa cells at 10 or 20 h after treatment (*n* = 8 llamas per time point for each treatment) from the **Experiment I** and at 10 or 20 h of in vitro culture from **Experiment II** (*n* = 4 independent replicates culture) using Trizol (Invitrogen, life Technology, USA) according to the manufacture’s recommendations. The purity of the samples was analyzed using the Nanodrop 1000 (Thermo Fischer Scientific, DE, USA). One microgram of total RNA was converted to complementary DNA (cDNA) using kit AffinityScript Q-PCR cDNA Synthesis (Agilent Technologies, Santa Clara, CA, USA) and Oligo-dT as per manufacturer’s instructions.

Brilliant III Ultra-Fast SYBR® Q-PCR master mix (Agilent, Santa Clara, California, USA) was used to detect and quantitate the transcripts. Samples were amplified in an Applied Biosystems® 7500 Q-PCR thermocycler using the following thermo cycle conditions: one cycle at 95 °C for 3 min, 40 cycles of 95 °C for 5 s, 60 °C for 30 s and 72 °C for 1 min. Data was analyzed using the thermocycler-associated software. For each sample cycle threshold values for the assayed transcripts were normalized for total input cDNA concentrations using cycle threshold values for transcripts for the “normalizer” house-keeping gene, Large ribosomal protein (RPLP0 [33]). The normalized values in each experiment were compared to a “calibrator” sample to determine the relative increase in the amount of the transcript. Primers for PCR were designed using primer express (PE Biosystems). To generate llama primers, sequences for *Vicugna pacos*, *Camelus Camelus ferus* (since *Vicugna pacos* sequence is not available for p450Aromatase gene) and *Bos taurus* were aligned for each gene of interest and primers were designed based on conserved sequences. Primer sets for transcript amplification were used at a final concentration of 250 nM each. Data was analyzed using the thermocycler-associated software. Additionally, following amplification the melting curves for the products was generated to ensure that the product represents a homogenous species. In addition, the PCR products were analyzed by electrophoresis to ensure that a product of the predicted size has been generated. The expected sizes of amplified products are shown in Table 1. Primers for P450scc, P450Arom, StAR and Large ribosomal protein (RPLP0) have already been tested and previously described for llama [27]. The mRNA relative expression of P450scc, P450Arom, StAR, HSD3B and VEGF were analyzed in Experiment I, whereas only StAR and VEGF were analyzed in Experiment II.

**Table 1:** Specific primer sequences to detect gene expression in llama in granulosa cells

Gene	ID	5′-3’	Length (bp)	product (bp)
3-beta-hydroxy steroid dehydrogenase (HSD3B)	LL-HSD3B-F	TGGTGGGCTTCTTGCTTAGT	20	174
	LL-HSD3B-R	CAACCCATTCCGTGGTATTC	20	
Steroidogenic acute regulatory protein (StAR)	LL-STAR-F	GAATGGGGACGAAGTGCTAA	20	200
	LL-STAR-R	GCTCATGGGTGATGACTGTG	20	
Side-Chain cleavage enzyme (p450scc)	LL-p450scc-F	GCCACTGCTCTTCCTGTCAT	20	237
	LL-p450scc-R	GCCATTTATTGCCTTCATGG	20	
Aromatase (p450 Arom)	LL-p450arom-F	GTGTCCGGAGTGTGCCTGTT	21	148
	LL-p450arom-R	GGAACCTGCAGTGGGAAATGA	21	
Vascular endothelial growth factor (VEGF)	LL-VEGF-F	TCCAATCGAGACCCTGGTAG	20	168
	LL-VEGF-R	ATCCGCATAATCTGCATGGT	20	
Large ribosomal protein (RPLP0)	rplp0-F	GGCGACCTGGAAGTCCAACT	20	149
	rplp0-R	CCATCAGCACCACAGCCTTC	20	

### Sequencing of llama steroidogenic and VEGF gene transcripts, partial mRNA

One micrograms of total RNA was reverse transcribed at 42 °C for 50 min using oligo dT 15 mer and SuperScript® II reverse transcriptase (Invitrogen, USA) to synthesize single-stranded cDNA. The PCR mixtures were prepared using a 5 μL of cDNA, 2,5 μL of 10X PCR buffer -Mg, 0,75 μL MgCl_2_ 50 mM, 0,5 μL of 10 mM dNTPs mix, 1 μL of 10 μM each specific primers and 2 units of Platinum™ *Taq* DNA polymerase (Invitrogen, USA) in a final reaction volume of 25 μL. The optimal PCR conditions were: 94 °C for 5 min; 42 cycles of 94 °C for 30s, 60 °C for 30s and 72 °C for 45 s; followed by a final step at 72 °C for 5 min. PCR products were separated on 2% agarose gels containing ethidium bromide (Invitrogen, USA). Amplicons of the expected size were purified using gel extraction kit E.Z.N.A (Omega Bio-Tek, Inc., USA). The sequencing reactions were performed with the reagent BigDye Terminator v3.1 Cycle Sequencing Kit (Applied Biosystems), following the manufacturer’s instructions and analyzed in ABI3500 Genetic Analyzer equipment (Applied Biosystems). Verification of homology analyzed by Clustal Omega (1.2.1) multiple sequence alignment.

### Statistical analysis

Progesterone/estradiol ratio of follicular fluid and progesterone concentration from primary culture of llama granulosa cells were analyzed among treatment groups by one-way analysis of variance (ANOVA). If significant differences were detected, means were compared among groups using Tuckey. Data for gene expression were analyzed by one-way analysis of variance (ANOVA), Dunnett test was used to compare all treatments with control group. Data for gene expression within treatment groups was analyzed using Student T Test. All data are reported as mean ± SEM. Analyses were performed using the Statistical Analysis System software package SAS Learning Edition, version 4.1 (SAS Institute, Inc., Cary, NC, USA, 2006).

## Results

### Target genes sequencing analysis

**Fig. 1:**
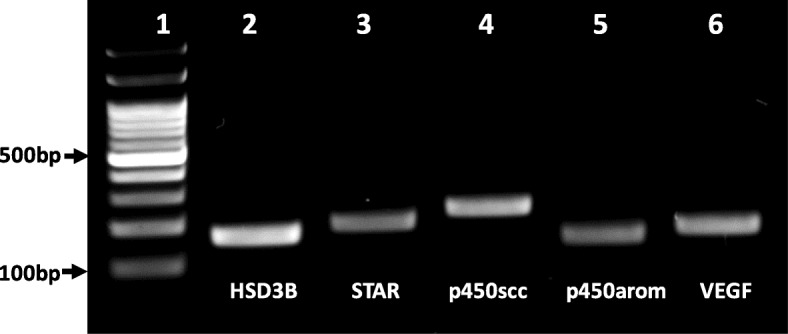
PCR amplifications products of steroidogenic enzymes and VEGF from llama granulosa cells

QPCR amplicons from granulosa cells matched the predicted *Vicugna pacos* P450scc, P450Arom, StAR, HSD3B and VEGF transcripts with 100% homology. The genes open reading frames consists of 200 (nt 447–646), 237 (nt 1604–1840), 148 (nt 281–428), 174 (nt 899–1072), 168 (nt 114–281) base pairs for StAR, P450scc, P450Arom, HSD3B and VEGF, respectively (Table 2, Fig. 1).

**Table 2:** Percentage of homology of steroroidogenic and VEGF transcripts from llama granulosa cells bands to *Vicugna pacos*

Gene	Identity	Gene Bank Accession N°	size (bp)	length of Llama Qpcr target (bp)	% of homology	start	stop
StAR	*Vicugna pacos* steroidogenic acute regulatory protein (STAR), mRNA	XM_006201129.1	1226	200	100	447	646
P450scc	*Vicugna pacos* cholesterol side-chain cleavage enzyme, mitochondrial-like (LOC102526586), mRNA	XM_006213724.1	1845	237	100	1604	1840
p450Arom	*Camelus ferus* aromatase (LOC102519005), mRNA	XM_006181316.2	1804	148	100	281	428
3ßHSD	*Vicugna pacos* 3 beta-hydroxysteroid dehydrogenase/Delta 5-- > 4-isomerase-like (LOC102528409), mRNA	XM_006216141.1	1122	174	100	899	1072
VEGF	*Vicugna pacos* vascular endothelial growth factor A (VEGFA), transcript variant X8, mRNA	XM_015237403.1	698	168	100	114	281

### Experiment I: in vivo *study*

**Fig. 2:**
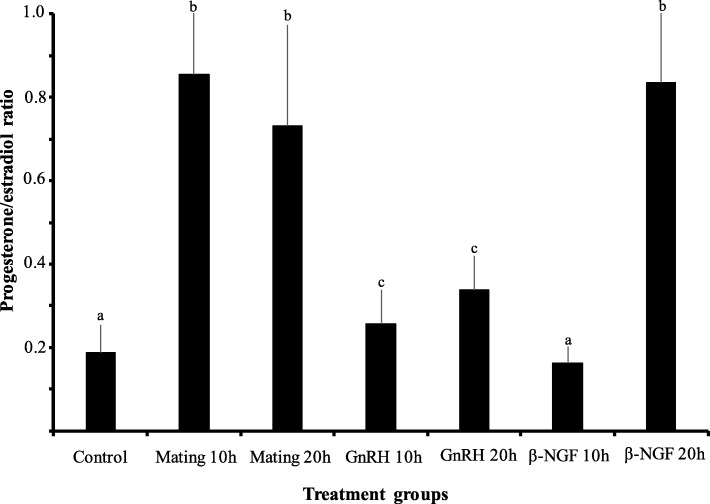
Preovulatory follicular fluid progesterone/estradiol ratio in llamas (mean ± SEM, *n* = 8/per treatment) at 10 and 20 h after natural mating or treatment with GnRH, β-NGF purified from llama seminal plasma or phosphate buffer saline (negative control group). Different superscripts, a, b, c, indicate significant differences among treatment groups (*P* < 0.01)

**Fig. 3:**
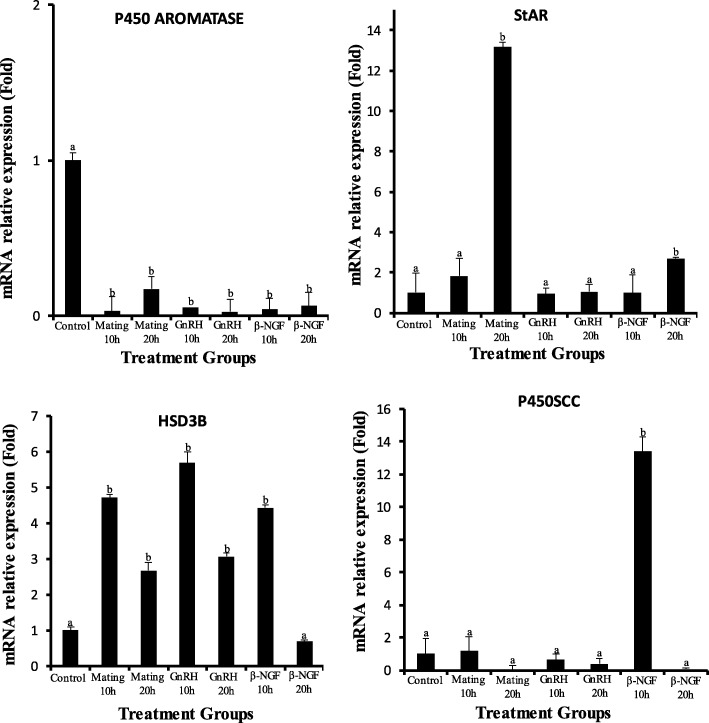
Relative mRNA abundance (mean ± SEM, *n* = 8/per treatment) of cytochrome CYP19A1/P450 aromatase, Star, HSD3B and CYP11A1/P450scc in llama preovulatory granulosa cells at 10 or 20 h after natural mating or treatment with GnRH, β-NGF purified from llama seminal plasma or phosphate buffer saline (negative control group). Different superscripts, a, b, c, indicate significant differences between control and the remaining groups (*P* < 0.01)

**Fig. 4:**
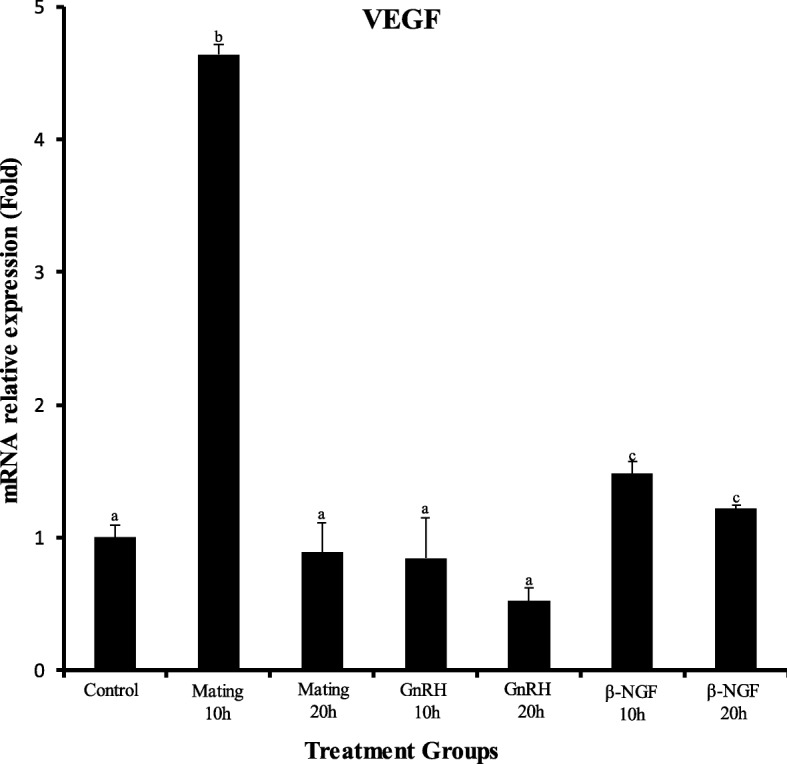
Relative mRNA abundance (mean ± SEM, *n* = 8/per treatment) of the angiogenic factor VEGF in llama preovulatory granulosa cells at 10 and 20 h after natural mating or treatment with GnRH, β-NGF purified from llama seminal plasma or phosphate buffer saline (control group). Different superscripts, a, b, c, indicate significant differences between control and the remaining groups (*P* < 0.01)

There was a significant increase in progesterone/estradiol ratio in mated llamas or females treated with GnRH or llama purified β-NGF. The greatest increase were observed in mated llamas at 10 and 20 h or in females treated with β-NGF at 20 h after treatment. An intermedia response was observed in GnRH-treated llamas with respect to the control group (Fig. 2). There was a significant down regulation in the mRNA expression of CYP19A1/P450 aromatase for both time points in llamas mated or treated with GnRH or llama purified β-NGF with respect to the control group. Additionally, there was an effect of treatment in the relative mRNA abundance of StAR, HSD3B and CYP11A1/P450scc. All treatments except β-NGF (20 h) significantly up-regulated the mRNA expression of HSD3B whereas the expression of StAR and CYP11A1/P450scc were significantly up-regulated only by mating (20 h), β-NGF (20 h) and β-NGF (10 h) respectively (Fig. 3). An interesting finding was that VGEF was up-regulated only in those llamas submitted to mating (10 h) or treated with purified β-NGF (10 and 20 h; Fig. 4).

**Fig. 5:**
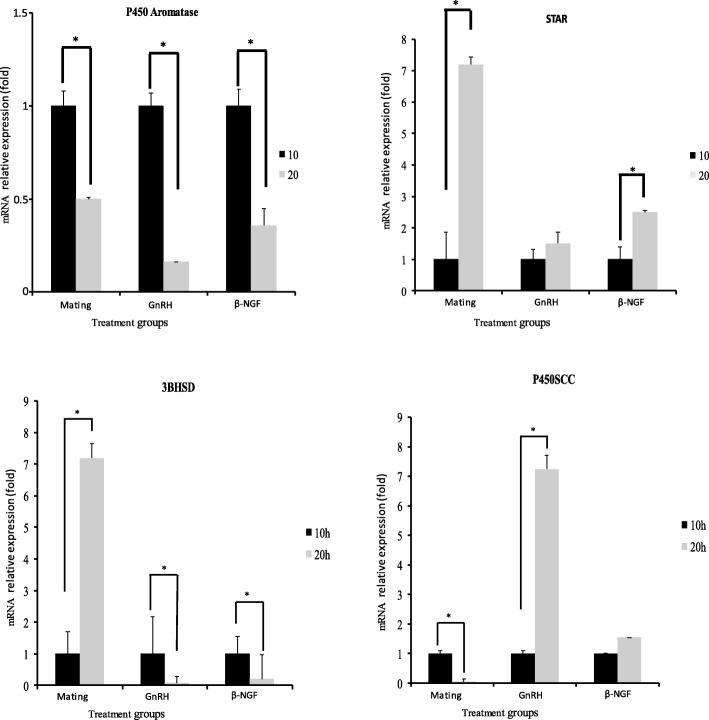
Relative mRNA abundance (mean ± SEM, *n* = 8/per treatment) of cytochrome CYP19A1/P450 aromatase, Star, HSD3B and CYP11A1/P450scc of llama preovulatory granulosa cells at 10 or 20 h after natural mating or treatment with GnRH or β-NGF purified from llama seminal plasma . * indicate significant differences within treatment groups (*P* < 0.01)

**Fig. 6:**
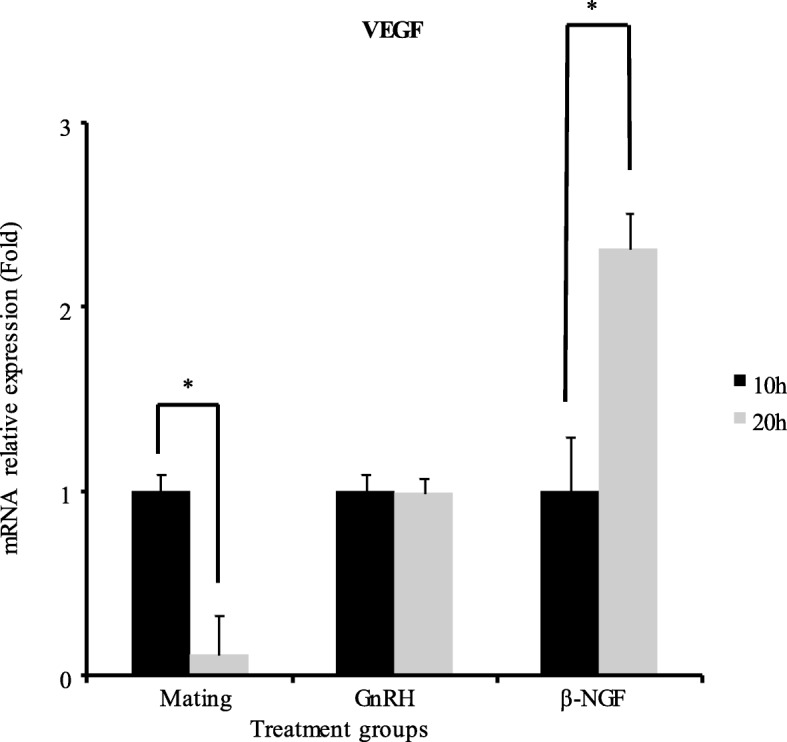
Relative mRNA abundance (mean ± SEM, *n* = 8/per treatment) of the angiogenic factor VEGF of llama preovulatory granulosa cells at 10 and 20 h after natural mating or treatment with GnRH or β-NGF purified from llama seminal plasma. * indicate significant differences within treatment groups (*P* < 0.01)

When comparing gene expression at 10 and 20 h within treatment groups, mRNA abundance of CYP19A1/P450 aromatase was down regulated, whereas, mRNA abundance StAR and HSD3B were significantly up regulated at 10 h in llamas submitted to mating whereas CYP11A1/P450scc was up regulated at 20 h in llamas treated with GnRH or β-NGF (Fig. 5). Additionally, VEGF mRNA abundance was significantly increased at 20 h after β-NGF administration (Fig. 6).

### Experiment II: in vitro *study*

**Fig. 7:**
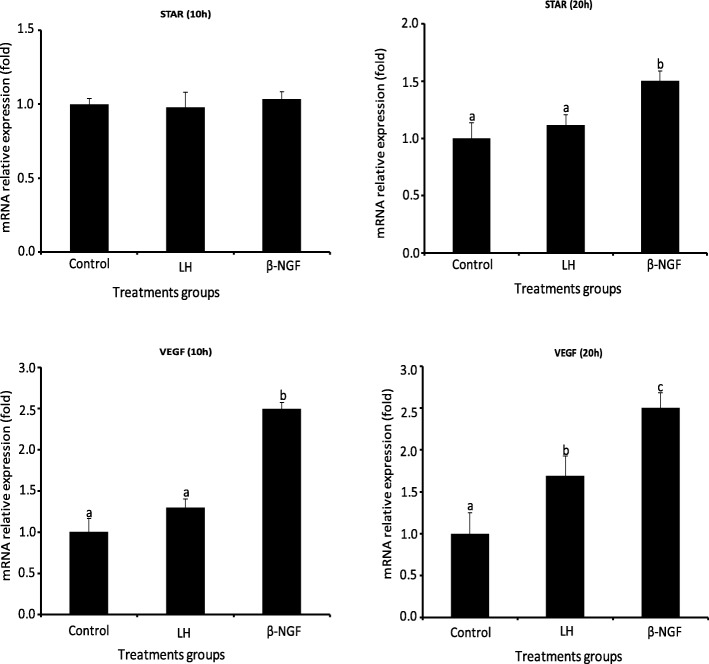
Relative mRNA abundance (mean ± SEM, *n* = 4 independent replicates culture) of StAR and the angiogenic factor VEGF from primary culture of llama granulosa cells at 10 or 20 h after 30 ng/ml of LH or 50 ng/ml of β-NGF purified from llama seminal plasma treatment. * indicate significant differences within treatment groups (*P* < 0.01)

This study was conducted to determine a local effect of LH and purified llama β-NGF on mRNA expression of StAR, as and VEGF and progesterone secretion from primary culture of llama granulosa cells collected from preovulatory follicles. The effect of LH or β-NGF treatment on mRNA expression of StAR and VEGF of llama granulosa cells are shown in Fig. 7.

Only β-NGF treatment induced an increase of mRNA abundance of StAR from llama granulosa cells at 20 h of in vitro culture, there was not effect of LH on StAR expression at any time point. There was a significant increase on mRNA abundance of VEGF at 10 and 20 h of in vitro *culture* from granulosa cells treated with β-NGF whereas LH treatment increases VEGF mRNA abundance only at 20 h of in vitro culture.

**Fig. 8:**
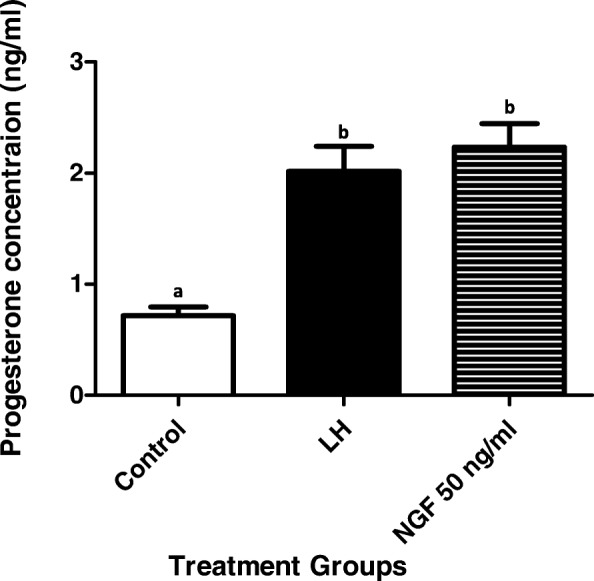
Progesterone secretion (mean ± SEM, *n* = 4 independent replicates culture) from primary culture of llama granulosa cells collected from preovulatory follicles and treated with 30 ng/ml of LH or 50 ng/ml of β-NGF purified from llama seminal plasma for 48 h. Different superscripts, a, b indicate significant differences among treatment groups (*P* < 0.01)

In addition, there was a significant increase on progesterone secretion from llama granulosa cells 48 h after LH or llama purified β-NGF treatment (Fig. 8).

## Discussion

A shift from estradiol to progesterone production pathway in the preovulatory follicle was induced in llamas after mating, or systemic administration of GnRH or purified β-NGF in the present study, as evidenced by an up-regulation of the main genes related to progesterone production resulting in an increase of P4/E2 ratio in the follicular fluid of preovulatory follicles.

The luteinization process begins after the LH surge and culminate long after ovulation occurs, resulting in the development of a Corpus Luteum. The first signs of the luteinization process take place in the granulosa and theca cells of the preovulatory follicle, where specifically granulosa cells switch form estradiol to progesterone secretion due to a dramatic change in the steroidogenic enzymes resulting in an increase in the progesterone/estradiol ratio in the follicular fluid [34, 35]. The LH surge induce a shift from estradiol to progesterone synthesis by triggering the transformation of granulosa and theca cells into large and small luteal cells, respectively [36]. It has been well described that progesterone concentration in the follicular fluid increases dramatically up to 4.5 fold already by 1.5 h after the LH surge induced by GnRH, returning to basal levels at 12 h with a second increase at 24 h after LH surge. Progesterone increase is accompanied with a dramatic decrease of estradiol production at 3.5 h after the LH surge [37–39].

Information about follicular P4/E2 ratio in induced ovulating species and more specifically in camelids is scarce. Changes in progesterone/estradiol ratio between small and large follicles has been described in the induced ovulating species, the dromedary [40–42]; however, no studies have been addressed to described the effect of the LH surge on follicular fluid steroids concentration and steroidogenic gene expression in camelids, and only limited in vivo and in vitro studies have been conducted in rabbit [43] and cat [44] respectively. Camelids as induced ovulatory species could be an interesting model to study periovulatory luteinization changes as the preovulatory LH surge is naturally induced by mating or chemical components of seminal fluid [45].

The P4/E2 ratio recorded in the preovulatory follicular fluid was affected by the treatments in the present study. The greatest increase in P4/E2 ratio was observed in mated llamas or those females given an intramuscular administration of 1.0 mg of purified β-NGF. Although, P4/E2 ratio did increase in llamas treated with GnRH, this rise was significantly lower than that observed after mating or β-NGF treatment. We have previously described the ovulatory and pituitary response in llamas after mating, or parenteral administration of GnRH or purified β-NGF [19, 20]. Interestingly, although ovulation rate did not differ among those treatments, the CL induced by mating, seminal plasma or β-NGF produced more progesterone than that of GnRH-treated females. Additionally, LH surge began earlier and it was more sustained in those females treated with seminal plasma or β-NGF resulting in a luteotrophic effect [7, 16]. Our results suggest that, the greater P4/E2 ratio induced by mating or β-NGF treatment during the periovulatory period could be associated to a better luteinization process in this species. On the other hand, it can not be ruled out that the greater increase in P4/E2 ratio induced by mating or β-NGF, could also be influenced by local effects of seminal components on granulosa cell function.

We have previously documented [45] that systemic β-NGF concentration significantly increases in llamas after natural mating or intrauterine infusion of seminal plasma. Although this β-NGF increase has been correlated to the preovulatory LH surge in the same study, we could not rule out a potential local role of β-NGF on ovarian function as it has been reported for other species [46, 47]. In fact, the addition of purified llama β-NGF in the in vitro culture of llama granulosa cells in the present study increases the mRNA relative expression of StAR at 20 h and VEGF at both 10 and 20 h after treatment whereas LH does not have any effect on StAR expression just on VEGF expression at 20 h after treatment. Nonetheless, it was interesting to find that primary in vitro culture of llama granulosa cells treated with LH are able to secret progesterone in the medium at similar concentration to those cells treated with β-NGF whereas progesterone concentration remained at basal levels in non-treated cells. A plausible explanation that LH did not affect StAR transcript expression in llama granulosa cells could be due to the time of 10 or 20 h considered to verify gene expression in the present study, it could not be the right window to determine the profile of the transcript. Perhaps, the expression of this gene is expressed before that period and all the expression was translated early in protein. It will be interesting to analyze HSD3B in in vitro granulosa cells culture in further studies to determine if LH up regulates this transcript at 10 or 20 h after treatment to find a plausible explanation regarding to the high progesterone secretion observed in llama granulosa cells treated with LH.

Interestingly, the high affinity receptor trkA is expressed in granulosa cells of pre antral and antral follicles in non-human primate ovary [48]. The presence of NGF and its high affinity receptor trkA has been found in granulosa cells of cows, sheep, rabbit and human [46, 49–52]. The receptor trkA is expressed in both thecal and granulosa cells (GCs) of bovine growing follicles [46], and freshly isolated in vitro culture theca cells from bovine antral follicles respond to NGF with androgen and progesterone secretion. Similar to those previous studies, the addition of purified llama β-NGF was able to increase progesterone production in the primary granulosa cell culture 48 h after treatment.

Changes in the expression patterns of the main steroidogenic enzymes after treatment give support to the observed shift in P4/E2 ratio in the follicular fluid. There was a significant down regulation of CYP19A1/P450 aromatase transcript in llamas submitted to mating or given GnRH or purified β-NGF in the present study suggesting a plausible switch of the steroidogenic pathways towards a progesterone-producing cell. Additionally, an up-regulation of transcripts StAR, HSD3B, CYP11A1/P450scc, related to progesterone synthesis, were observed at different time points (10 or 20 h) after different ovulation stimulatory treatments. These findings are in agreement with the results reported in rodents, primates and ruminants [35, 53, 54], where the increase of the mRNA abundance of HSD3B, StAR, CYP11A1/P450scc and the decrease of CYP19A1/P450 aromatase induced by LH or hCG administration were consistent with conversion of granulosa cells from primarily estrogen to progesterone producing cells.

Interestingly, the transcript HSD3B was up regulated in all groups at 10 h after treatment administration whereas StAR and CYP11A1/P450scc were up regulated only by mating or purified β-NGF administration. These results suggest that regulation of these last two genes could be differentially affected by the LH profile induced by mating or β-NGF, or by local effects of seminal components on llama granulosa cells steroidogenesis. Similarly, up regulation of StAR and CYP11A1/P450scc transcripts was observed in a previous llama study [27] where intramuscular administration of purified β-NGF was able to increase by 2.0 fold the mRNA abundance of CYP11A1/P450scc in luteal cells at day 4 and 10 of CL development, and 2.0 fold for StAR by day 4. However, the same genes were not affected in those llamas treated with GnRH, given support to the notion that seminal β-NGF, in association with the increase in the circulating concentrations of LH or as a single local effect, exerts also ovarian local influences on steroidogenesis.

Interestingly, when comparing gene expression patterns within treatment groups, mRNA abundance of CYP19A1/P450 aromatase is down regulated and StAR and HSD3B were significantly up regulated by 20 h in mated llamas, whereas in females treated with GnRH or β-NGF, during the same time-frame, these genes were down regulated. These findings suggest that seminal components, different from NGF, may have a role in the enhancement of the expression of these genes during the crucial hours of the periovulatory period. However, those same factors may have had an opposite effect regarding the expression of CYP11A1/P450scc, which was up regulated at 20 h only in llamas treated with GnRH or β-NGF.

Additionally to changes in steroidogenic gene expression, there was an up regulation of VEGF transcript only in mated llamas and those treated with purified β-NGF, however VEGF was not affected by GnRH treatment. Interestingly the increase of this angiogenic factor could be related to a previous llama study [23] where using Power Doppler ultrasonography we demonstrated that the preovulatory follicle and the early CL have a greater vascularization area in those females treated with purified β-NGF than that of GnRH-treated llamas, resulting in a higher progesterone secretion. Indeed, further studies confirmed that the luteotrophic effect of purified β-NGF was associated with the enhancement of tissue vascularization during the preovulatory period and early stages of CL development [23, 24]. It has been also reported that NGF promotes ovarian angiogenesis by enhancing the secretion of VEGF through the MAPK-Erk1/2 activation pathway of tyrosine kinase trkA receptor in human granulosa cells [55]. Therefore, an increase of the vascular endothelial growth factor (VEGF) which induces angiogenesis by stimulating the proliferation of endothelial cells of preexisting capillaries could have a pivotal role in the enhancement of ovarian steroidogenesis [56].

These observations are reinforced when comparing VEGF gene expression within treatments groups, since VEGF mRNA abundance was significantly increased by 20 h after β-NGF intramuscular administration, an effect not observed in mated or GnRH treated llamas.

To the best of our knowledge this is the first in vivo characterization of hormonal and gene expression changes in preovulatory follicles in the llama model after treatments that induce an LH surge, demonstrating a rapid shift towards progesterone at the expense of estradiol production, similar to what has been observed in spontaneous ovulating species [31] and also in rabbits [38]. Moreover, our results strongly suggest that in this animal model not only the preovulatory LH surge, but also seminal components such as β-NGF, trigger changes in angiogenic factors and steroidogenic enzymes gene expression that induce periovulatory changes in the follicle fluid P4/E2 ratio. Interestingly, the increase in progesterone concentration observed in the fluid of large diameter follicles in dromedaries has been correlated to final stages of oocyte in vivo maturation [36]. If LH surge induced by mating or GnRH differentially influences oocyte nuclear and cytoplasmic maturation in llamas and alpacas warrants further investigation.

## Conclusions

Based on the results of the present study, we can conclude that natural mating and systemic administration purified llama β-NGF from seminal fluid induce a rapid shift from estradiol to progesterone production pathway in the preovulatory follicle in llamas; as evidenced by an up-regulation of the main genes related to progesterone production resulting in an increase of P4/E2 ratio in the follicular fluid. Differences in gene expression patterns of steroidogenic enzymes between GnRH and mated or β-NGF-treated llamas suggest local effects of seminal components, which could be direct or associated with LH increase as evidenced for the effect of purified llama β-NGF on StAR and VEGF expression in primary llama granulosa cell culture. Finally, the angiogenic factor VEGF was significant up regulated after β-NGF treatment at all-time points reinforcing previous observations of B-NGF stimulated increase in preovulatory follicle vascularization.

## Data Availability

All data generated in the present study are available from the corresponding author on reasonable request.

## Abbreviations

ANOVA: Analysis of variance
CL: Corpus Luteum
GC: Granulosa cells
HSD3B: 3β -hydroxysteroid dehydrogenase
P4/E2: Progesterone/estradiol ratio
P450 aromatase or CYP19A1: cytochrome P450 aromatase
P450scc or CYP11A1: cytochrome P450 side-chain cleavage
RPLP0: Large ribosomal protein
StAR: Steroidogenic acute regulatory protein
VEGF: Vascular Endothelial Growth Factor
β-NGF: Beta Nerve growth factor
